# Effectiveness of Mobile Technology in Managing Fatigue: Balert App

**DOI:** 10.3389/fpsyg.2021.704955

**Published:** 2021-07-21

**Authors:** Ricardo De La Vega, Héctor Anabalón, Cristian Jara, Eduardo Villamil-Cabello, Miguel Chervellino, Álvaro Calvo-Rodríguez

**Affiliations:** ^1^Department of Physical Education, Sport & Human Movement, Autonomous University of Madrid, Madrid, Spain; ^2^AlertPlus, Santiago, Chile; ^3^Escuela Universitaria Don Bosco, Madrid, Spain; ^4^University of A Coruña, A Coruña, Spain

**Keywords:** mental fatigue, heart rate variability, stroop, laboral fatigue, APP

## Abstract

The performance of professional tasks with a high cognitive, emotional, and even physiological demand, can cause a state of mental fatigue, which implies attentional alterations, greater errors in the tasks performed and a decrease in personal and work productivity caused by a deterioration of the cognitive control processes. The present study presents a mobile phone application named BAlert that allows monitoring and controlling the body's fatigue processes based on the scores obtained in the Stroop effect and the heart rate variability. A pilot study has been carried out with a sample of 63 adults who have used the application a total of 942 times. The results allow us to classify the subjects, by logistic regression analysis, in their fatigue levels in 74% of the occasions. These results highlight the importance of this mobile application to control work fatigue processes in different possible scenarios (military, health, sports, business, etc.).

## Introduction

Fatigue, either central or peripheral, has become one of the most important research topics throughout decades (de la Vega et al., 2021). Besides, the analysis of psychophysiological fatigue is considered very important in different contexts (Lohani et al., 2019) resulting in studies related to human's response to external and internal loads (Wijesuriya et al., 2007; Wilson et al., 2007). The external loads exerted on the individual are added to their skills and coping strategies, leading to a level of tolerance and adaptation to each situation (Folkman and Lazarus, 1988). Along the last decades, there has been shown differences between physical and mental fatigue roles, highlighting clear limitations in the study of central fatigue due to its indirect ways of measuring it, which emphasizes the importance of developing new central fatigue analysis procedures (Bittner et al., 2000). On the contrary, in order to measure physiological fatigue, there have been indicated clear methodologies (de la Vega et al., 2021).

One of the main and most studied areas involving fatigue is physical activity and sports performance (Rampinini et al., 2008). Those physically fatiguing activities, such as team sports, also demand cognitive efforts, as it requires sustained attention to make accurate and quick decisions sustained by processing information from a changing environment (Walsh, 2014). According to these demands, sports may also produce mental fatigue (understood as a psychobiological state characterized by tiredness and lack of energy produced by demanding and sustained periods of cognitive activity (Marcora et al., 2009; Smith et al., 2016). This mental fatigue combined with the implicit physical fatigue usually results in a reduction of performance (Pageaux et al., 2015; Smith et al., 2016). It is also important to assess the impact of mental fatigue on decision-making skills because it is well-known in the literature that decreases cognitive performance resulting in a negative impact during laboratory-controlled tests on decision-making (Pageaux and Leppers, 2018). Stoop effect can be defined as the inference between two stimuli with different levels of relevance that are happening at the same time (Banich, 2019) which has no single locus on the brain. This could be useful to measure fatigue.

In the occupational and labor field, different technological applications are being developed that allow analyzing the response of workers to demands and requirements. Given the important role of sleep debt and the effect on cognitive performance as a relevant factor of fatigue (Belenky and Akersted, 2011), mathematical models, techniques and procedures have been developed, measuring performance in the operating environment and clinical practice to reduce risks of poor performance, lost productivity, errors, incidents and accidents in the workplace and play a crucial role in managing the risk of fatigue to improve performance, productivity, safety, and in the long run, term, improve the health and well-being of workers (Thomas et al., 2006; Van Dongen et al., 2010). Certain detection technologies may be helpful in determining both types of fatigue, but technology aimed at increasing alertness may only be suitable for countering TR fatigue. Technologies currently exist which enable detection of driver fatigue and interventions that have the potential to dramatically reduce crash probability (May and Baldwin, 2009). The successful implementation of these technologies depends on the cause and type of fatigue experienced (Begum et al., 2012; He et al., 2013).

As we know, fatigue is a function of the interaction of multiple factors, including the background of the sleep/wake cycle, the circadian rhythm phase and the workload, being modulated by individual differences in response to these factors (Van Dongen et al., 2005). A fatigue-inducing factor is one that shifts the distribution of fatigue risk in the direction of increasing the risk of error, incident or accident as is the case with the workload and cognitive performance People vary from each other in their sensitivity to these factors. This relative variability in sensitivity to sleep loss appears to be a lasting individual trait (Belenky and Akersted, 2011).

Fatigue measurements is operationally defined by subjective self-assessment and objectively by decreased alertness that reflects lower performance. The fatigue self-report consists of a verbal response (for example, the subject says “I'm tired”) or a written response responding to a fatigue scale. The lower performance can also be measured by a variety of forms such as cognitive test and others (Thomas et al., 2006).

There are individual differences in response to factors that cause fatigue which could have an association with genetic markers. There are also age-related differences as older people perform less than younger people when both are in rest, but perform better than younger people when both have sleep restrictions. In addition, there are individual differences which are likely to affect fatigue measured by self-perception and objective measures.

As we have presented, detection of fatigue could be very useful. However, there are very few reliable Apps that can provide a suitable tool for that purpose. Even some cutting edge technologies, such us eye-tracker, have proven that are useful classifying mental fatigue (Li et al., 2020), but it demands much more materials than a simple smartphone.

Certain detection technologies may be helpful in determining both types of fatigue, but technology aimed at increasing alertness may only be suitable for countering TR fatigue.

In this sense, virtual platforms and applications for evaluation and research will provide a useful tool to obtain individual subjects and collect all the information (Hernández-Mendo and y González-Ruiz, 2012; Huberty et al., 2019). Technologies currently exist which enable the detection of driver fatigue and interventions that have the potential to dramatically reduce the crash probability. The successful implementation of these technologies depends on the cause and type of fatigue experienced.

The main goal of this research is to analyze the usefulness of the BeAlert application for the detection of fatigue processes through the measurement of heart rate variability and the values obtained in the Stroop test.

## Materials and Methods

This study followed a quasi-experimental design (Montero and León, 2007) and it received the approval of the University Ethical Commission in compliance with the Helsinki Declaration. All subjects were informed about the procedure and gave their written consent to participate. This study was carried out complying with the Standards for Ethics in Sport and Exercise Science Research (Harriss et al., 2019).

### Participants

The participants included 63 individuals from Madrid, Spain, 20 of whom were female and 43 male. The participants were aged between 18 and 42 years (*M*_years_ = 23.62, *SD*_years_ = 4.87). All of the participants regularly engaged in physical activity, between 4 and 14 h per week (*M*_hours/week_ = 8.15, *SD*_hours/week_ = 3.02), BMI between 16.4 and 31.4 (*M*_BMI_ = 23.18, *SD*_BMI_ = 2.4); and number of hours aware between 0 and 10 (*M*_hoursaware_ = 4.4, *SD*_hoursaware_ = 3.01). Six participants were excluded from the study for not completing the measurements correctly. Intentional sampling methods were used (Montero and León, 2007). Participants were asked to complete the mobile app task at least once a day for a period of 1 week. The total data collected was N = 942 (N_female_ = 285; N_male_ = 657).

### Instrumentation and Study Variables

The number of hours of physical activity per week and the scores obtained on the DT test were used as controlled variables. This allows us to have a relatively similar population in the variable “level of physical activity,” which can affect the fatigue results obtained in the mobile app. Therefore, only the subjects in which there were no statistically significant differences in their weekly level of physical exercise were used in our pilot study (de la Vega et al., 2021).

To carry out this research, a mobile application (AP Móvil), has been created based on the application of two independent tests: (i). The Stroop effect, which allows the evaluation of the speed in making decisions simple (identify “same color—same word” -e.g.: “the green word written in green letters); and complex (identify “color—word of another color “e.g.: “the green word written in red”). The number of correct responses and the reaction time to the appearance of the stimuli on the screen are taken into account; and (ii). The temporal parameters of the Heart Rate Variability (AVNN, RMSSD, SDNN, and PNN50). The app is available for download on Android and IOS systems (https://play.google.com/store/apps/details?id=net.alertplus.amfsmovil&hl=es_419&gl=US). The engineering team implemented both tests with the previous considerations offered to be able to properly record the values of both tests. The application allows the registration of all the values that, later, are used to offer the results on fatigue to the participants.

The duration of this test was 5 min approximately. In addition, the values of the body mass index and the number of hours awake were taken.

### Procedure

All participants voluntarily participated in the research. An informational meeting was held where participants completed their informed consent. Instructions were offered to all volunteers so that they could download the application on their mobile phones, explaining its use. In order to carry out this pilot study, they were asked to carry out the test at least once a day for a period of 2 weeks.

### Statistics

We have used the Multinomial Logistic Regression Analysis (MLRA), which allows us to analyze the relationship between multiple explanatory variables and different categorical variables (Schulte et al., 2003). It has been decided that group 1 (Fast STROOP and low SDNN) is the reference group on which the statistics of the rest of the groups will be analyzed (Fast STROOP and High SDNN; Slow STROOP and Low SDNN; Slow STROOP and High SDNN). The dependent variable used is composed of deviation in SDNN score, as a result of subtracting the SDNN of each person with respect to the theoretical SDNN that they should have according to their age (Nunan et al., 2010). From this variable, we can therefore interpret values >0 as High SDNN, and values <0 as low SDNN. On the other hand, we have the variable “Average STROOP Milliseconds” as the average time it takes each person to click for each of the 35 stimuli that were presented. For the purpose of this research, values <500 ms were interpreted as fast STROOP, and values ≥500 ms were interpreted as slow STROOP.

The classification variables based on the dependent variable are the scores offered in the mobile app in terms of stress level (S), RMSSD, AVNN, perceived quality of food (PQF), gender (male, female), body mass index (BMI) and the number of hours awake (HA). The global fit of the model was checked with the Nagelkerke R-squared statistic (Nagelkerke, 1991). All computations are carried out using the R Commander statistical language (see R Project http://cran.r-project.org). The significance level was set at *p* < 0.05.

## Results

The global fit of the model was checked with the Nagelkerke R-squared statistic with a value of 0.81 and with the classification table that shows a classificatory success rate of 74%. Both indicators reflect a very good fit of our logistic regression model with the chosen variables.

Descriptive statistics are presented in Tables 1, 2. The descriptive statistics values of each of the study variables are presented, as well as the grouping variables used.

**Table 1:** Summary of descriptive data.

**Term**	**No. of studies**	**Range**	**Mean**	**SD**
SDNN (ms)	942	0–137	55.2	27.4
RMSSD (ms)	942	0–207	54.4	32.9
STRESS (Amo/2VRMo)	846	0–1,745	194.9	206.1
Age (years)	942	17–69	23	9.4
BMI (kg/m^2^)	942	16.4–31.4	23.1	2.4
AVNN (ms)	942	0–1,487	844.7	284.9
HR (bpm)	942	40–107	67.8	12.3
Hours awake	942	0–10	4.4	3
Epworth score	942	0–14	5.9	3.4

**Table 2:** Summary of descriptive data of SDNN-Stroop distribution.

	**Frequency**	**Valid percentage**	**Accumulated percentage**
Fast stroop—Low SDNN	186	21.3	21.3
Fast stroop—High SDNN	370	42.3	63.5
Slow stroop—Low SDNN	150	17.1	80.7
Slow stroop—High SDNN	169	19.3	100.0
Total	875	100.0	

SD = standard deviation; SDNN = standard deviation of normal to normal intervals; RMSSD = root mean square of successive differences; BMI = body mass index; Amo = mode amplitude—% intervals corresponding to Mode; VR = variational range—difference between min and max r-r intervals; Mo = mode—most frequent R-R interval value.

In the Table 3, all the independent explanatory variables of the model are presented in the first column. The order, from highest to lowest importance for the model as a whole, is as follows: (1). Stress; (2). Age; (3). RMSSD; (4). HR; (5). AVNN; (6). Food; (7). Female; (8). Epworth; (9). BMI; and (10). Hours awake.

**Table 3:** Results from multivariate logistic regression model containing all explanatory variables (full model).

**Fast stroop—Low SDNN**	**β estimate**	**Standard error**	**Sig**
Stress (*β_0_*)	−0.048	0.95	0.000
Age 17–21 (*β_1_*)	−10.454	0	0.000
Age 22–30 (*β_2_*)	−8.515	0	0.000
RMSSD (*β_3_*)	0.065	1.07	0.000
HR (*β_4_*)	0.417	1.52	0.000
AVNN (*β_5_*)	0.019	1.02	0.000
Alimentation (*β_6_*)	0.431	1.54	0.143
Female (*β_7_*)	−0.303	0.74	0.503
Epworth (*β_8_*)	−0.162	0.85	0.009
BMI (*β_9_*)	0.335	1.40	0.000
Hours awake (*β_10_*)	0.054	1.06	0.373
**Slow stroop—Low SDNN**	***β*** **estimate**	**Standard error**	**Sig**
Stress (*β_0_*)	−0.001	1.00	0.999
Age 17–21 (*β_1_*)	−4.208	0.01	0.000
Age 22–30 (*β_2_*)	−4.75	0.01	0.000
RMSSD (*β_3_*)	0.009	1.01	0.529
HR (*β_4_*)	0.227	1.25	0.002
AVNN (*β_5_*)	0	1.00	0.857
Alimentation (*β_6_*)	−0.922	0.40	0.000
Female (*β_7_*)	0.227	1.25	0.535
Epworth (*β_8_*)	0.196	1.22	0.000
BMI (*β_9_*)	0.12	1.13	0.062
Hours awake (*β_10_*)	0.051	1.05	0.302
**Slow stroop—High SDNN**	***β*** **estimate**	**Standard error**	**Sig**
Stress (*β_0_*)	−0.043	0.96	0.000
Age 17–21 (*β_1_*)	−10.317	0.00	0.000
Age 22–30 (*β_2_*)	−10.222	0.00	0.000
RMSSD (*β_3_*)	0.069	1.07	0.000
HR (*β_4_*)	0.377	1.46	0.000
AVNN (*β_5_*)	0.016	1.02	0.000
Alimentation (*β_6_*)	−0.843	0.43	0.004
Female (*β_7_*)	1.325	3.87	0.003
Epworth (*β_8_*)	−0.033	0.97	0.585
BMI (*β_9_*)	0.29	1.34	0.001
Hours awake (*β_10_*)	−0.065	0.94	0.303

The interpretations summarize the meaning of each exponent of the β: how much the probability of occurrence of the event increases or decreases given a score in each independent variable (ceteris paribus).

The global fit of the model was checked with the Nagelkerke R-squared statistic with a value of 0.81 and with the classification table that shows a classificatory success rate of 74%. Both indicators reflect a very good fit of our logistic regression model with the chosen variables. Based on the odd ratios and Wald's statistic, the order of importance of the independent variables is: (i). stress; (ii). Age; (iii). RMSSD; (iv). HR; (v). AVNN; (vi). Food; (vii). gender (female); (vii). Epworth; (viii). BMI.

The profiles that maximize the probability for each group are: (i). Person older than 30 years, low STRESS, low RMSSD, high HR, high AVNN, low EPWORTH, and high BMI for fast STROOP and low SDNN; (ii). Person older than 30 years, with high HR, low alimentation and high EPWORTH for slow STROOP and low SDNN. (iii). Women, older than 30, low STRESS, high RMSSD, high HR, high AVNN, low alimentation and high BMI for slow STROOP and high SDNN; and (iv). Men between 17 and 21 years, high STRESS, low RMSSD, low HR, low AVNN, high alimentation, and low BMI for fast STROOP and low SDNN.

## Discussion

Given the poor subjective perception of the level of fatigue, it is advisable to emphasize the establishment of objective parameters in the assessment (Shahid et al., 2010). For a more objective profile, various psychometric evaluations have been used, such as the PVT, the WAFA and the Stroop test, among others. In addition, the incorporation of biometric measurements that evaluate the psychophysiological status such as heart rate variability (HRV) has been very useful as a marker of fatigue and has been an element used in the evaluation of stress and acute mental load in workers from various areas and also as a relevant element in the detection of driver fatigue (Castaldo et al., 2015; Fallahi et al., 2016; Abtahi et al., 2018; Digiesi et al., 2020) The relationship between the variability of the heart rate of the people evaluated with sleep debt has also been described (Heinze et al., 2011). We use this evaluation together with objective elements such as the evaluation of the cognitive level with the Stroop Test and in this way the structure of the Balert device is organized to include the fundamental variables responsible for the development of fatigue.

Early detection of mental fatigue could be very practical in order to prevent occupational hazard and it has also importance in productivity (Li et al., 2020) in different sectors such as education, sports, the military context or the professions that must manage fatigue situations such as drivers. In the present study, a Stroop task had to be carried out with a large proportion of congruent trials. This large proportion of congruent trials paradoxically increases working memory load (Kane and Engle, 2003), since congruent trials encourage goal neglect such that the goal of naming the color has to be actively maintained in working memory and cannot be automatized during the time course of the task. Due to this complication, the task was more cognitively demanding than tasks used previously in the analysis of mental fatigue; in addition, since the Stroop task has also been employed in studies of self-control loss, an analysis of the time evolution of this loss of cognitive control was possible.

Finally, it should be noted that it would be of interest to weight all these average time results in the Stroop taking into account the success/error of the person clicking on each color, and check if the results change by weighing these successes/errors in the average time of the Stroop. A study on Stroop should be sought and consulted with which to replicate the construction of an indicator that summarizes the temporal information of the Stroop relativized with the rate of successes/errors of people in each click.

## Data Availability Statement

The datasets presented in this article are not readily available because right now the development company owns the rights and is in the development phase. Requests to access the datasets should be directed to hector.anabalon@alertplus.cl.

## Ethics Statement

The studies involving human participants were reviewed and approved by Autonomous University of Madrid. The patients/participants provided their written informed consent to participate in this study.

## Author Contributions

RD: planning of the research design, statistical analysis, analysis of results, and writing of the article. HA: app development, research design planning, and article writing. CJ and MC: design and app development. EV-C: planning of the research design, analysis of results, and writing of the article. ÁC-R: statistical analysis. All authors contributed to the article and approved the submitted version.

## Conflict of Interest

HA, CJ, and MC were employed by company AlertPlus. The remaining authors declare that the research was conducted in the absence of any commercial or financial relationships that could be construed as a potential conflict of interest.

## References

[B1] Abtahi F. Anund A. Fors C. Seoane F. Lindecrantz K. (2018). Association of Drivers' sleepiness with heart rate variability: A Pilot Study with Drivers on Real Roads in EMBEC & NBC 2017. EMBEC 2017, NBC 2017. IFMBE Proceedings, vol 65, eds. Eskola H. Väisänen O. Viik J. Hyttinen J. (Singapore: Springer). 10.1007/978-981-10-5122-7_38

[B2] Banich M. (2019). The stroop effect occurs at multiple points along a cascade of control: evidence from cognitive neuroscience approaches. Front. Psychol. 10:2164. 10.3389/fpsyg.2019.02164 31681058 PMC6797819

[B3] Begum S. Ahmed M. U. Funk P. Filla R. (2012). Mental state monitoring system for the professional drivers based on Heart Rate Variability analysis and Case-Based Reasoning, in Federated Conference on Computer Science and Information Systems (FedCSIS), 35–42.

[B4] Belenky G. Akersted T. (2011). Occupational sleep medicine, in Principals and Practice of Sleep Medicine (St. Louis, MO: Elsevier Saunders), 734–737. 10.1016/B978-0-444-53817-8.00012-8

[B5] Bittner R. Hána K. Poušek L. Smrka P. Schreib P. Vysoký P. (2000). Detecting of fatigue states of a car driver, in Medical Data Analysis. ISMDA 2000. Lecture Notes in Computer Science, Vol. 1933, eds Brause R. W. Hanisch E. (Berlin; Heidelberg: Springer). 10.1007/3-540-39949-6_32

[B6] Castaldo R. Melillo P. Bracale U. Caserta M. Triassi M. Pecchia L. (2015). Acute mental stress assessment via short term HRV analysis in healthy adults: a systematic review with meta-analysis. Biomed. Signal Proces. 18, 370–377. 10.1016/j.bspc.2015.02.012

[B7] de la Vega R. Jiménez-Castuera R. Leyton-Román M. (2021). Impact of weekly physical activity on stress response: an experimental study. Front. Psych. 11:608217. 10.3389/fpsyg.2020.608217 33510685 PMC7835705

[B8] Digiesi S. Manghisi V. M. Facchini F. Klose E.M. Foglia M.M. Mummolo C. (2020). Heart rate variability based assessment of cognitive workload in smart operators. Manage. Prod. Eng. Rev. 11, 56–64. 10.24425/mper.2020.134932

[B9] Fallahi M. Motamedzade M. Heidarimoghadam R. Soltanian A. R. Miyake S. (2016). Assessment of operators' mental workload using physiological and subjective measures in cement, city traffic and power plant control centers. Health Promot. Perspect. 6, 96–103. 10.15171/hpp.2016.17 27386425 PMC4932229

[B10] Folkman S. Lazarus R. S. (1988). Coping as a mediator of emotion. J. Pers. Soc. Psychol. 54, 466–475. 10.1037/0022-3514.54.3.466 3361419

[B11] Harriss D. J. Macsween A. Atkinson G. (2019). Ethical standards in sport and exercise science research: 2020 update. Int. J. Sports Med. 40, 813–817. 10.1055/a-1015-3123 31614381

[B12] He J. Roberson S. Fields B. Peng J. Cielocha S. Coltea J. (2013). Fatigue detection using smartphones. J. Ergon. 3:3. 10.4172/2165-7556.1000120

[B13] Heinze C. Trutschel U. Edwards D. Sirois B. Golz M. (2011). Asymmetric properties of heart rate variability to assess operator fatigue, in Proceedings of the Sixth International Driving Symposium on Human Factors in Driver Assessment, Training and Vehicle Design. Olympic Valley - Lake Tahoe, California (Iowa City, IA: Public Policy Center, University of Iowa), 475-481. 10.17077/drivingassessment.1435

[B14] Hernández-Mendo A. Morales-Sánchez V. y González-Ruiz S. L. (2012). A virtual platform for on-line evaluation and research: MenPas. Cuader. Psicol Deporte 12, 147–150. 10.4321/S1578-84232012000100015

[B15] Huberty J. Eckert R. Larkey L. Joeman L. Mesa R. (2019). Experiences of using a consumer-based mobile meditation app to improve fatigue in myeloproliferative patients: qualitative study. JMIR Cancer 5:e14292. 31333197 10.2196/14292 PMC6681641

[B16] Kane M. J. Engle R. W. (2003). Working-memory capacity and the control of attention: The contributions of goal neglect, response competition, and task set to Stroop interference. J. Exp. Psychol. 132, 47–70. 10.1037/0096-3445.132.1.47 12656297

[B17] Li J. Li H. Umer W. Wang H. Xing X. Zhao S.. (2020). Identification and classification of construction equipment operators' mental fatigue using wearable eye-tracking technology. Automat. Construct. 109:3000. 10.1016/j.autcon.2019.103000

[B18] Lohani M. Payne B. R. Strayer D. L. (2019). A review of psychophysiological measures to assess cognitive states in real-world driving. Front. Hum. Neurosci. 13:57. 10.3389/fnhum.2019.00057 30941023 PMC6434408

[B19] Marcora S. M. Staiano W. Manning V. (2009). Mental fatigue impairs physical performance in humans. J. Appl. Physiol. 106, 857–864. 10.1152/japplphysiol.91324.2008 19131473

[B20] May J. F. Baldwin C. L. (2009). Driver fatigue: the importance of identifying causal factors of fatigue when considering detection and countermeasure technologies. Transp. Res. Part F Traffic Psych. Behav. 12, 218–224. 10.1016/j.trf.2008.11.005

[B21] Montero I. León O. G. (2007). A guide for naming research studies in psychology. Int. J. Clin. Health Psychol. 7, 847–862. Available online at: https://www.redalyc.org/articulo.oa?id=33770318

[B22] Nagelkerke N. (1991). A note on a general definition of the coefficient of determination. Biometrika 78, 691–692. 10.1093/biomet/78.3.691

[B23] Nunan D. Sandercock G. R. Brodie D. A. (2010). A quantitative systematic review of normal values for short-term heart rate variability in healthy adults. Pacing Clin Electrophys. 33, 1407–1417. 10.1111/j.1540-8159.2010.02841.x 20663071

[B24] Pageaux B. Leppers R. (2018). The effects of mental fatigue on sport-related performance. Progr. Brain Res. 240, 291–315. 10.1016/bs.pbr.2018.10.004 30390836

[B25] Pageaux B. Marcora S. Rozan V. Leppers R. (2015). Mental fatigue induced by prolonged self-regulation does not exacerbate central fatigue during subsequent whole-body endurance exercise. Front. Hum. Neurosci. 9:67. 10.3389/fnhum.2015.00067 25762914 PMC4340216

[B26] Rampinini E. Impellizzeri F. M. Castagna C. Azzalin A. Bravo D. F. Wisloff U. (2008). Effect of match-related fatigue on short-passing ability in young soccer players. Med. Sci. Sports Exerc. 40, 934–942. 10.1249/MSS.0b013e3181666eb8 18408603

[B27] Schulte A. Sweet S. A. Grace-Martin K. (2003). Data analysis with SPSS: a first course in applied statistics. Teach. Sociol. 31:126. 10.2307/3211435

[B28] Shahid A. Shen J. Shapiro C. M. (2010). Measurements of sleepiness and fatigue. J. Psychos. Res. 60, 81–89. 10.1016/j.jpsychores.2010.04.001 20630266

[B29] Smith M. R. Zeuwts L. Lenoir M. Hens N. De Jong L. Coutts A. J. (2016). Mental fatigue impairs soccer-specific decision-making skill. J. Sports Sci. 34, 1297–1304. 10.1080/02640414.2016.1156241 26949830

[B30] Thomas M. Sadlier M. Smith A. (2006). The effect of Multi Convergent Therapy on the psychopathology, mood and performance of Chronic Fatigue Syndrome patients: A preliminary study, Counselling and Psychotherapy Research, 6, 91–99, 10.1080/14733140600711955

[B31] Van Dongen H. Vitellaro K. Dinges D. F. (2005). Individual differences in adult human sleep and wakefulness: leitmotif for a research agenda. Sleep 28, 479–496. 10.1093/sleep/28.4.479 16171293

[B32] Van Dongen S. Wijnaendts L. C. D. ten Broek C. Galis F. (2010). Human fetuses and limb asymmetry: No evidence for directional asymmetry and support for fluctuating asymmetry as a measure of developmental instability. Anim. Biol. 60, 169–182. 10.1163/157075610X491716

[B33] Walsh V. (2014). Is sport the brain's biggest challenge? Curr. Biol. 24, R859–R860. 10.1016/j.cub.2014.08.003 25247362

[B34] Wijesuriya N. Tran Y. Craig A. (2007). The psychophysiological determinants of fatigue. Int. J. Psychophysiol. 63, 77–86. 10.1016/j.ijpsycho.2006.08.005 17007946

[B35] Wilson G. F. Caldwell J. A. Russell C. A. (2007). Performance and psychophysiological measures of fatigue effects on aviation related tasks of varying difficulty. Int. J. Aviat. Psychol. 17, 219–247. 10.1080/10508410701328839

